# STAT3 Activation in Skeletal Muscle Links Muscle Wasting and the Acute Phase Response in Cancer Cachexia

**DOI:** 10.1371/journal.pone.0022538

**Published:** 2011-07-20

**Authors:** Andrea Bonetto, Tufan Aydogdu, Noelia Kunzevitzky, Denis C. Guttridge, Sawsan Khuri, Leonidas G. Koniaris, Teresa A. Zimmers

**Affiliations:** 1 Sylvester Comprehensive Cancer Center, University of Miami Miller School of Medicine, Miami, Florida, United States of America; 2 Department of Cell Biology and Anatomy, University of Miami Miller School of Medicine, Miami, Florida, United States of America; 3 Center for Computational Science, University of Miami Miller School of Medicine, Miami, Florida, United States of America; 4 Human Cancer Genetics, Department of Molecular Virology, Immunology and Medical Genetics, Ohio State University School of Medicine, Columbus, Ohio, United States of America; 5 Division of Surgical Oncology, DeWitt Daughtry Family Department of Surgery, University of Miami Miller School of Medicine, Miami, Florida, United States of America; 6 Division of Burns, DeWitt Daughtry Family Department of Surgery, University of Miami Miller School of Medicine, Miami, Florida, United States of America; McMaster University, Canada

## Abstract

**Background:**

Cachexia, or weight loss despite adequate nutrition, significantly impairs quality of life and response to therapy in cancer patients. In cancer patients, skeletal muscle wasting, weight loss and mortality are all positively associated with increased serum cytokines, particularly Interleukin-6 (IL-6), and the presence of the acute phase response. Acute phase proteins, including fibrinogen and serum amyloid A (SAA) are synthesized by hepatocytes in response to IL-6 as part of the innate immune response. To gain insight into the relationships among these observations, we studied mice with moderate and severe Colon-26 (C26)-carcinoma cachexia.

**Methodology/Principal Findings:**

Moderate and severe C26 cachexia was associated with high serum IL-6 and IL-6 family cytokines and highly similar patterns of skeletal muscle gene expression. The top canonical pathways up-regulated in both were the complement/coagulation cascade, proteasome, MAPK signaling, and the IL-6 and STAT3 pathways. Cachexia was associated with increased muscle pY705-STAT3 and increased STAT3 localization in myonuclei. STAT3 target genes, including SOCS3 mRNA and acute phase response proteins, were highly induced in cachectic muscle. IL-6 treatment and STAT3 activation both also induced fibrinogen in cultured C2C12 myotubes. Quantitation of muscle versus liver fibrinogen and SAA protein levels indicates that muscle contributes a large fraction of serum acute phase proteins in cancer.

**Conclusions/Significance:**

These results suggest that the STAT3 transcriptome is a major mechanism for wasting in cancer. Through IL-6/STAT3 activation, skeletal muscle is induced to synthesize acute phase proteins, thus establishing a molecular link between the observations of high IL-6, increased acute phase response proteins and muscle wasting in cancer. These results suggest a mechanism by which STAT3 might causally influence muscle wasting by altering the profile of genes expressed and translated in muscle such that amino acids liberated by increased proteolysis in cachexia are synthesized into acute phase proteins and exported into the blood.

## Introduction

Cachexia, or progressive wasting of fat and skeletal muscle despite adequate nutrition, is a pervasive and devastating complication of cancer [1], [2], [3]. Cachexia afflicts more than half of all cancer patients and results in weakness, diminished quality of life, poor response to therapy, and susceptibility to illness. Moreover, cachexia itself is responsible for 25–30% of all cancer-related deaths [1], [2]. Currently, there are no approved, effective treatments for muscle wasting in cancer.

Clinically, cancer cachexia is defined as weight loss of at least 5% in the presence of underlying illness with associated muscle weakness, fatigue, anorexia, low lean body mass and abnormal biochemistry, including increased inflammation, anemia and low serum albumin. Weight loss of 5%, 10% or 15% total body weight is referred to as mild, moderate or severe cachexia, respectively, and both weight loss and the rate of weight loss correlate positively with mortality [4]. The etiology of cachexia is multi-factorial. Although a subset of patients experience early satiety and anorexia, studies have demonstrated that nutritional intake in cachectic patients should be sufficient to maintain body weight, but they lose weight regardless [5]. As well, cachectic patients can be hypo-, normo-, or hyper-metabolic with respect to resting energy expenditure, suggesting that alterations of metabolic rate alone cannot be responsible for the observed loss of body mass [2]. Furthermore, tumor competition for metabolic fuels is an unsatisfactory explanation of cachexia, both because pinpoint tumors can produce cachexia and because human tumors of 500 g or larger do not necessarily induce wasting [6].

The systemic metabolic derangements noted in cancer cachexia are also observed with other forms of systemic inflammation [7]. Cytokines, including Tumor Necrosis Factor (TNF)/cachectin, interleukin (IL)-1α, IL-1β, interferon-γ, and IL-6 have been implicated in cachexia both through experimental manipulation in mouse models and by association of serum levels in patients with cachexia [6], [7]. IL-6 is a multifunctional cytokine involved in a variety of host defenses and pathological processes [8]. Others and we have shown that IL-6 administration to mice is sufficient to induce wasting of muscle and fat stores and in the most severe cases, ultimately death [9], [10], [11], [12], [13], [14]. As well, IL-6 plays a substantial role in inducing cachexia in mice bearing the colon-26 cancer cell line and the uterine cancer line, Yomoto, as administration of IL-6 blocking agents reduce muscle wasting in those models [15], [16], [17], [18]. Serum IL-6 is a sensitive predictor of weight loss, including in patients with advanced small cell lung cancer [19] and colon cancer [20]. IL-6 and other gp130 ligands such as LIF and CNTF are thought to mediate cachexia through a combination of anorexia, lipid catabolism, insulin resistance, and effects on protein synthesis and degradation. However, the molecular pathways regulated by IL-6 in skeletal muscle that lead to muscle wasting are unknown.

IL-6 acts on cells by binding the IL-6 receptor α-chain (IL-6Rα), also known as gp80, either in its membrane-bound or soluble form, inducing dimerization of gp130 and activation of its associated Janus kinases (JAKs), which tyrosine phosphorylates gp130 [21]. These events result in the activation of two major signaling pathways, STAT3, and the mitogen-activated protein kinase (MAPK/ERK) cascade. STAT3 activation by tyrosine phosphorylation leads to dimerization and nuclear translocation, DNA binding and modulation of gene expression. In cooperation with c-Jun and various forms of the transcription factor CAAT enhancer-binding protein (C/EBP), IL-6 signaling through STAT3 up-regulates acute phase response protein gene expression in the liver. The acute phase response is a generalized response of the innate immune system, consisting of some 40 genes up-regulated up to several thousand-fold upon stimulation [22].

Tisdale and Fearon with co-workers have linked cancer cachexia with persistent elevations in acute phase response proteins in serum [23], [24], [25]. Approximately 50% of all cancer patients demonstrate increased serum acute phase response proteins at the time of cancer diagnosis, and the percentage of cancer patients with an elevated serum acute phase response increases with disease burden and stage [24], [26]. Elevated levels of fibrinogen are observed in many malignancies, including lung and melanoma, and increased expression correlates with a bleak outcome [27], [28]. Current models posit that tumor and host derived factors induce proteolysis in order to fuel a chronic hepatic acute phase response. Production of acute phase response proteins in the liver requires catabolism of structural proteins in skeletal muscle as a source of amino acids. Given the differences in amino acid utilization in muscle proteins versus acute phase reactants, up to 2.6 g of muscle protein or 12 g of muscle tissue may be wasted to synthesize 1 g acute phase response proteins [29].

We sought to determine potential relationships between elevated cytokines, the acute phase response and wasting in cancer. Here we report characterization of the serum cytokines and the muscle transcriptome in the colon-26 adenocarcinoma model of cancer cachexia. We provide evidence of STAT3 activation, target gene expression and the acute phase response in both liver and skeletal muscle in these mice, providing a molecular link between these observed phenomena.

## Results

### Colon26 model of cancer cachexia

**Figure 1:**
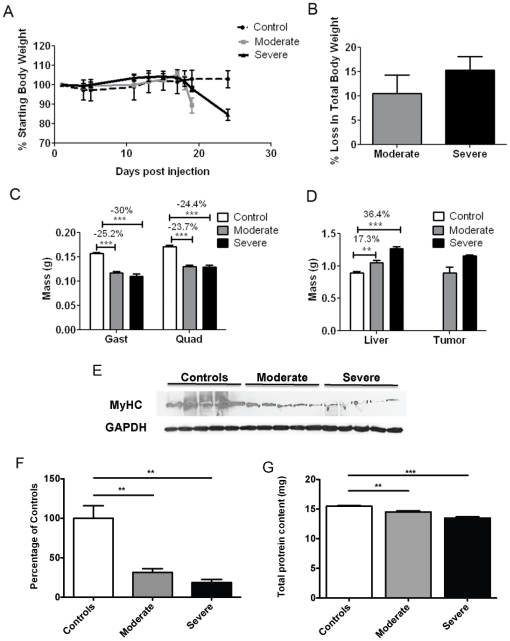
Characterization of C26 cachexia. A, B, Body weight changes for control mice and for mice injected with C26 tumor cells. Mice euthanized on day 19 had lost ∼10% body weight, considered moderate cachexia. Mice euthanized on day 24 had lost ∼15% body weight, considered severe cachexia. C, Significantly decreased quadriceps and gastrocnemius weights were observed with C26 cachexia. Differences were not significant in muscles from moderate versus severe cachexia. D, Liver weight and tumor weight both increased with severity/duration of cachexia. E, Representative Western blotting for Myosin Heavy Chain (MyHC) in quadriceps muscle from both control and tumor bearing mice. F, Densitometric quantification of the Western blotting for MyHC protein shows a marked reduction in both moderate (−69% vs. controls) and severe (−81% vs. controls) cachexia. G, Total protein content in quadriceps muscle from control and tumor-bearing mice. Protein content is significantly reduced in both moderate (−7% vs. controls) and severe (−13% vs. controls). n = 4–5 per group; **P<0.01, ***P<0.001.

Implantation of colon-26 (C26) adenocarcinoma cells into Balb/c or CD2F1 mice is a classic model of cancer cachexia [30], [31]. As in most cancer patients, food intake is not reduced in C26 cachexia and IL-6 levels are elevated [30]. Interventions that reduce IL-6 in this model also inhibit wasting [15], [16], [17], [18]. In our hands, CD2F1 female mice injected with C26 cells experienced progressive declines in total body weight (Figure 1A-B). Mice were sacrificed at 10% weight loss (19 days after injection) and 15% weight loss (24 days), reflecting moderate and severe cachexia. The decline in total body weight was accompanied by a proportionately greater loss of muscle mass (Figure 1C). Quadriceps weight decreased 23.7% ( *p*<0.001 vs. PBS-injected controls) in moderately cachectic mice and 24.4% ( *p*<0.001) in severely cachectic mice. Similarly, gastrocnemius muscle weights declined 25.2% ( *p*<0.001) in moderate cachexia and 30.0% ( *p*<0.001) in severe cachexia. Such declines in muscle mass are known to result from increased protein degradation due to an activation of the proteolytic machinery [32] as well as from loss of specific muscle proteins, such as Myosin Heavy Chain (MyHC) [33]. Consistent with a loss in muscle protein, soluble quadriceps protein content (that measured in standard protein extracts) was reduced in both disease states (Figure 1G). Moreover, MyHC was significantly reduced in both moderate (−69%, p<0.01) and severe (−81%, p<0.01) cachexia (Figure 1E–F).

In contrast to muscle wasting, total liver mass increased 17.3% ( *p*<0.01) in moderate cachexia and 36.4% (*p*<0.001) in severe cachexia (Figure 1D). Tumor size also increased with time and wasting (Figure 1D), as did spleen size (data not shown).

Plasma analyte profiling of both moderately and severely cachectic mice revealed that circulating levels of IL-6 and three other gp130 ligands, IL-11, leukemia inhibitory factor (LIF) and Oncostatin M were significantly increased in tumor-bearing mice (Table 1). Levels of Tumor Necrosis Factor (TNF), IFN-γ and IL-1α, three other cytokines frequently associated with cachexia, were significantly elevated as well. Serum levels of the IL-6 responsive acute phase reactants fibrinogen, haptoglobin and Von Willebrand Factor were also markedly induced in response to the tumor. Unlike in humans, C Reactive Protein (CRP) is not an acute phase protein in mice and was not elevated in cachexia. This elevation of plasma IL-6 and the acute phase response proteins, combined with the increase in liver mass are consistent with the known effects of IL-6 in mediating the hepatic acute phase response [13], [34], [35], [36], [37]. These results in mice with C26 tumors parallel the association of the IL-6 and acute phase response with muscle wasting in cancer patients.

**Table 1:** Increased serum cytokines and acute phase response proteins with Colon-26 cachexia.

	Normal	Moderate Cachexia	Severe Cachexia
**gp130 Ligands**			
IL-6 (pg/ml)	<LOW>	104±41***	120±22***
IL-11 (pg/ml)	<LOW>	2010±122***	3180±1424*
LIF (pg/ml)	1570±170	1997±165*	2137±172*
OSM (ng/ml)	<LOW>	0.121±0.060*	0.220±0.024***
**Other cachexia-associated cytokines**			
TNF (ng/ml)	<LOW>	0.063±0.006*	0.049±0.005*
IL-1α (pg/ml)	62±17	204±90	214±0***
IFN-γ (pg/ml)	<LOW>	29±7.7**	15±8**
**Acute phase proteins**			
Fibrinogen (µg/ml)	30,800±6,350	165,333±21,455**	159,667±58,586***
Haptoglobin (µg/ml)	17±1.3	164±11.5***	173±20.1***
Von Willebrand factor (ng/ml)	75±36.7	322±49.7***	359±65.6**

*P<0.05.

**P<0.01.

***P<0.001 versus normal. No differences between moderate and severe cachexia were statistically significant. <LOW> indicates below the level of detection.

### Transcription profile of the C26 model of cancer cachexia

**Figure 2:**
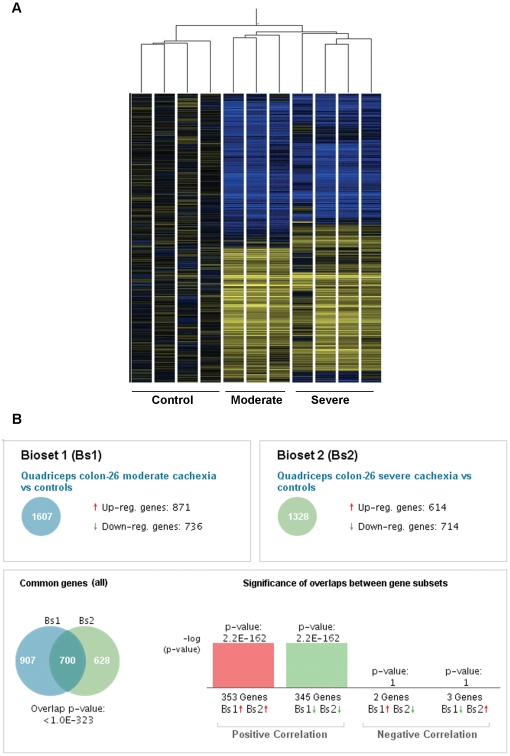
Overlapping patterns of gene expression in skeletal muscle in C26 cachexia. A, Heat map of quadriceps gene expression in C26 cachexia showing distinct clustering of genes by experimental groups. Samples are normalized to the controls. Blue indicates down-regulated genes, yellow up-regulated genes, and black no change. Only genes with *P*<0.05 by one-way ANOVA are shown. B, Comparison of gene expression in moderate and severe C26 cachexia (NextBio).

We sought to identify changes in muscle gene expression associated with wasting. Microarray analysis and data normalization was performed on RNA isolated from the quadriceps of mice with moderate or severe C26 cachexia and non-tumor bearing controls. Unsupervised hierarchical classification of the samples clearly distinguished control muscle, moderately wasted and severely wasted samples (Figure 2A).

Statistical analysis was performed to identify genes exhibiting significant changes in expression during cachexia. Among 45,281 transcripts, 1607 genes were found to be differentially expressed in moderate cachexia and 1328 in severe wasting (–2≤ fold change ≥2, *p*<0.05) (Figure 2B). Overall, 700 genes were expressed in common in moderate and severe cachexia, of which all but 5 were regulated in the same direction in both groups (Table S2). The top 30 genes by fold-change regulated in each of moderate and severe cachexia are shown in Tables 2 and 3. Of the remainder, 907 were unique to moderate cachexia (Table S3) and 628 to severe cachexia (Table S4). Similarity of gene expression between the two groups was highly significant ( *p* = 1.0 e-323). Despite the many significantly changed genes, most genes commonly considered to be “housekeeping” genes and used for normalization strategies were not different among groups. Specifically, gylceraldehyde-3-phosphate dehydrogenase (GAPDH), beta-actin (ACTB), b-actin (ACTB), 18S RNA (RN18S), aldolase (ALDOA, ALDOB, ALDOC) and most tubulins (TUBA2, TUBB1, TUBB2a, TUBB2c, TUBB3, TUBB4, TUBG1, TUBG) were unchanged.

**Table 2:** Top 30 genes regulated in quadriceps in moderate C26 cachexia.

Gene	Gene Description	p-value	Fold Change
Fga	fibrinogen alpha chain	0.0007	61.0
Itih3	inter-alpha trypsin inhibitor, heavy chain 3	0.0003	59.2
Otop1	otopetrin 1	5.50E-05	−59.2
Egln3	EGL nine homolog 3	7.40E-05	55.6
Ucp1	uncoupling protein 1 (mitochondrial, proton carrier)	0.0176	−44.1
Anxa13	annexin A13	0.0158	34.8
Pdzd7	PDZ domain containing 7	0.002	−34.7
Trim7	tripartite motif-containing 7	0.0017	−31.3
Saa1	serum amyloid A 1	0.008	29.9
Cxxc6	tet oncogene 1	0.0243	−29.6
Ntrk3	neurotrophic tyrosine kinase, receptor, type 3	0.001	−29.6
Il1r2	interleukin 1 receptor, type II	0.0068	26.6
Doc2b	double C2, beta	0.0075	25.6
Snf1lk	salt inducible kinase 1	0.001	25.4
Apoa1	apolipoprotein A–I	0.0124	24.5
Mmd2	monocyte to macrophage differentiation-associated 2	0.0059	−24.4
Aldh1a7	aldehyde dehydrogenase family 1, subfamily A7	0.0044	−24.4
Ambp	alpha 1 microglobulin/bikunin	0.0239	22.1
Aqp4	aquaporin 4	0.0352	−21.1
Scgb3a1	secretoglobin, family 3A, member 1	0.0014	21.1
Dynlrb1	dynein light chain roadblock-type 1	6.40E-05	−20.6
Lypd6	LY6/PLAUR domain containing 6	0.0365	−18.9
Cxcl13	chemokine (C-X-C motif) ligand 13	0.0162	18.6
Tmem118	ring finger protein, transmembrane 2	0.0369	−18.5
Scg3	secretogranin III	6.20E-05	−18.3
Plcd4	phospholipase C, delta 4	0.0284	−17.9
Lrrc38	leucine rich repeat containing 38	0.016	−17.3
Maff	v-maf musculoaponeurotic fibrosarcoma oncogene family, protein F	0.0068	17.2
Hpx	hemopexin	0.0018	17.1

**Table 3:** Top 30 genes regulated in quadriceps in severe C26 cachexia.

Gene	Gene Description	p-value	Fold Change
Nnat	neuronatin	1.40E-05	−96.2
Cilp2	cartilage intermediate layer protein 2	0.0002	−39.4
Lcn2	lipocalin 2	0.007	33.1
Saa1	serum amyloid A 1	0.0107	33.0
Gm1611	gene model 1611, (NCBI)	0.0015	−32.9
Itih3	inter-alpha trypsin inhibitor, heavy chain 3	0.0004	29.9
Scgb3a1	secretoglobin, family 3A, member 1	0.0007	29.1
Rsph1	radial spoke head 1 homolog (Chlamydomonas)	1.50E-09	−28.4
Cxcl13	chemokine (C-X-C motif) ligand 13	0.0086	24.0
Lypd6	LY6/PLAUR domain containing 6	0.0044	−22.2
Aqp4	aquaporin 4	0.0024	−21.1
Myoz3	myozenin 3	0.008	−20.6
Plcd4	phospholipase C, delta 4	0.0389	−20.4
Actc1	actin, alpha, cardiac muscle 1	0.0004	−19.4
Kcng4	potassium voltage-gated channel, subfamily G, member 4	0.0006	−18.4
Rapgef6	Rap guanine nucleotide exchange factor (GEF) 6	0.0099	−18.1
Serpina3m	serine (or cysteine) peptidase inhibitor, clade A, member 3M	0.0058	16.2
Csf2rb2	colony stimulating factor 2 receptor, beta 2, low-affinity (granulocyte-macrophage)	0.0285	15.7
D0H4S114	DNA segment, human D4S114	0.0004	−15.7
Itih4	inter alpha-trypsin inhibitor, heavy chain 4	0.0001	15.7
Vsig4	V-set and immunoglobulin domain containing 4	0.0138	15.45
Mmd2	monocyte to macrophage differentiation-associated 2	0.0358	−15.4
Il1r2	interleukin 1 receptor, type II	0.0173	15.2
Pld5	phospholipase D family, member 5	0.0068	−13.9
Mt2	metallothionein 2	0.0184	13.9
Adamts4	a disintegrin-like and metallopeptidase (reprolysin type) with thrombospondin type 1 motif, 4	0.0003	13.0
Serpina3n	serine (or cysteine) peptidase inhibitor, clade A, member 3N	0.0048	13.0
Gdap1	ganglioside-induced differentiation-associated-protein 1	0.0181	−12.6
3526401B18Rik	RIKEN cDNA 3526401B18 gene	0.0048	−12.6
Chad	chondroadherin	0.0023	−12.6

### Known muscle growth regulatory genes

**Figure 3:**
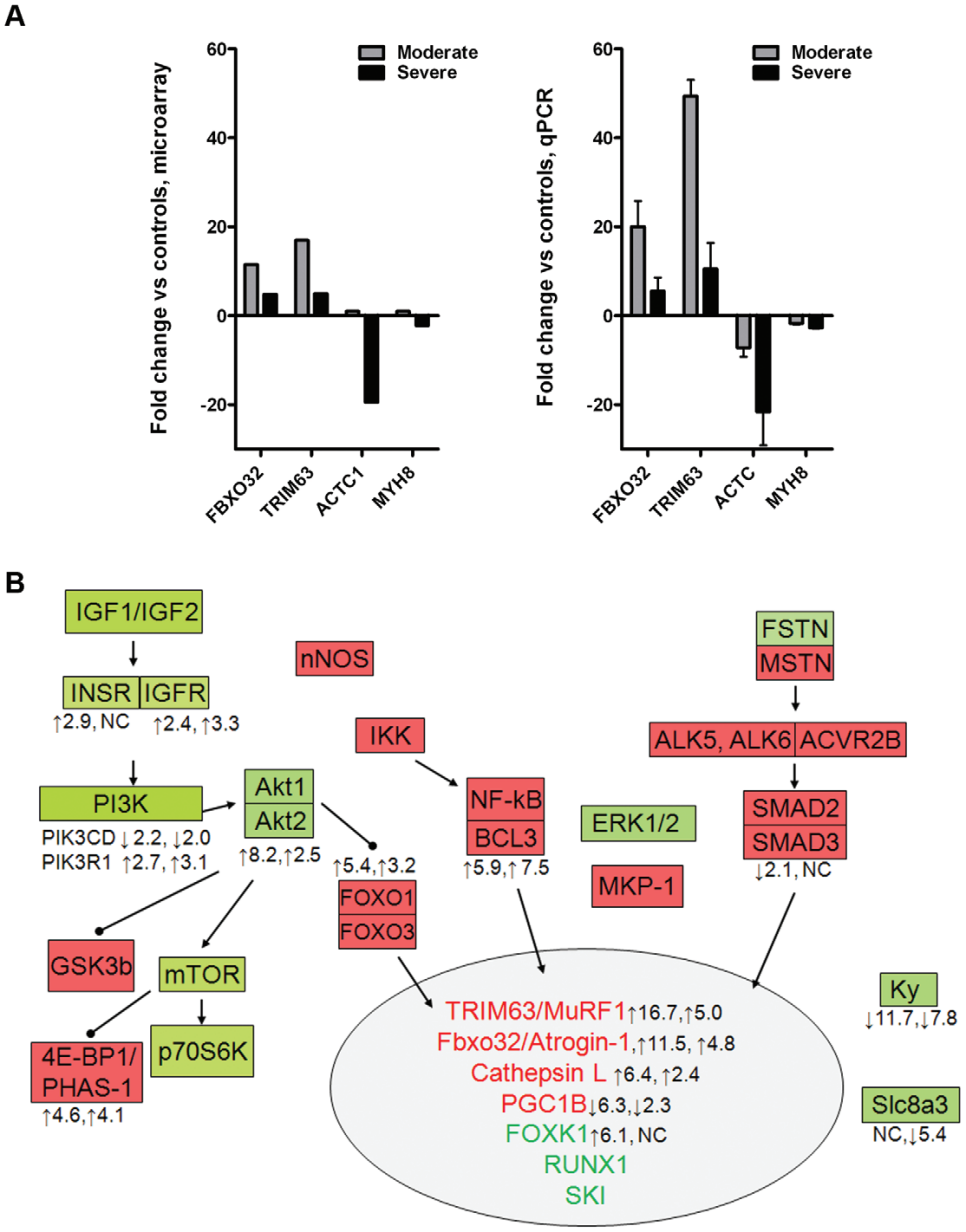
Expression of known genes in muscle growth regulation in C26 cachexia. A, Expression of the ubiquitin ligases, Fbxo32/Atrogin-1 and Trim63/MuRF1 are induced by microarray and qPCR analysis. Expression of genes encoding muscle structural proteins, including ACTA1 and MHY8 are decreased. B, Gene expression patterns in C26 cachexia mapped onto a schematic of muscle growth regulation pathways. Genes with demonstrated growth-promoting activity in muscle are shown in green, growth inhibitory genes in red. The number on the left represents fold-change in moderate cachexia and right in severe cachexia, with the arrow indicating the direction of the change. Genes with no values were either not changed or not present in the dataset.

Muscle wasting of virtually all etiologies is associated with increased expression of skeletal muscle ubiquitin E3 ligases, Trim63/Murf-1 and Fbxo32/Atrogin-1 [38], [39], [40]. Both were markedly increased in our microarray analysis, with a greater increase in Trim63/Murf-1 versus Fbxo32/Atrogin-1 and higher expression in moderate versus severe wasting (Figure 3). These trends were confirmed by quantitative real-time RT-PCR. Muscle atrophy is also associated with reduced expression of RNAs encoding structural proteins. Indeed, by microarray and qRT-PCR, cardiac alpha actin and myosin heavy chain 8 were down regulated, more so in severe versus moderate wasting. These results are consistent with reports from others in C26 cachexia [33].

### Pathway analysis

**Figure 4:**
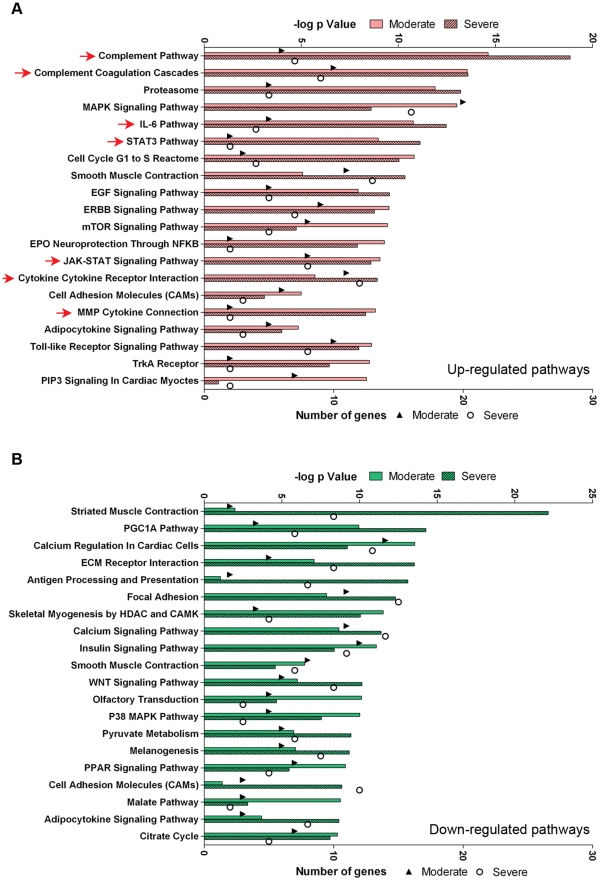
C26 cachexia pathway analysis. A. The top 20 Broad MSigDB canonical pathways significantly up-regulated in moderate and severe cachexia versus controls. Pink bars (upper axis) represent the significance of the overlap. The triangles (moderate cachexia) and circles (severe cachexia) (lower axis) denote the number of genes differentially expressed in that pathway. Red arrows indicate pathways related to IL-6/STAT3/inflammation. B, The top 20 Broad MSigDB canonical pathways significantly down-regulated in moderate and severe cachexia versus controls. Green bars (upper axes) represent the significance of the overlap. The triangles (moderate cachexia) and circles (severe cachexia) (lower axis) as above.

In order to identify molecular pathways regulated in response to C26-induced cachexia, gene profiles from quadriceps of control, moderate and severe cachexia were analyzed using NextBio (Figure 4). Of the top 20 Broad MSigDB canonical pathways up-regulated in both moderate and severe colon-26 cachexia, eight were related to inflammation, including the complement and coagulation pathways, IL-6, STAT3, JAK-STAT, cytokine and Toll-like receptor pathways. Pathways related to the proteasome, mitogen activated protein kinase (MAPK), the cell cycle, muscle contraction, epidermal growth factor, ErbB and mTOR signaling were also increased.

The top canonical pathways down-regulated were related to skeletal muscle contraction, peroxisome proliferator-activated receptor-γ coactivator-1α, calcium regulation, extracellular matrix interactions, skeletal myogenesis, and insulin and WNT signaling, among others.

### STAT3 activation and target gene expression in C26 cachexia

**Figure 5:**
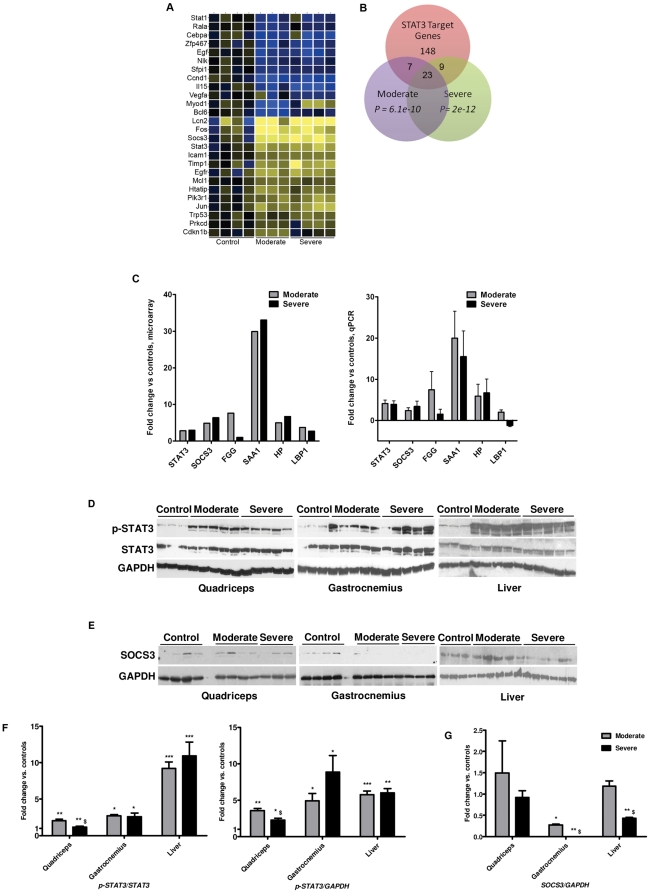
STAT3 and its target genes are activated in muscle in C26 cachexia. A: Heat map of gene expression changes of Stat3 and co-cited gene products, as identified by Genomatix Bibliosphere. Blue indicates down regulated genes, yellow up regulated, and black no change. Only genes with P<0.05 by one-way ANOVA are shown. B: A subset of STAT3 target genes identified through the literature are differentially regulated in moderate and severe cachexia versus controls. C: STAT3 and its target genes SOCS3 and CEBPD are increased at the mRNA level by microarray and qPCR. D: Protein levels for p-STAT3 and STAT3 in protein extracts from quadriceps, gastrocnemius and liver evaluated by Western blotting analysis. E: SOCS3 protein levels. F: quantitative analysis of p-STAT3/STAT3 and p-STAT3/GAPDH ratio (expressed as fold-change vs. controls). G: quantitative analysis of SOCS3 protein levels (expressed as fold-change vs. controls). GAPDH was used as an internal reference to confirm equal loading. n = 3–5 per group; *P<0.05, **P<0.01, ***P<0.001 vs. Controls, ^$^P<0.05 vs. moderate.

Given the prominence of the cytokine/IL-6/STAT3 pathways in the microarray analysis, we sought to characterize expression of STAT3 interacting genes in our model. Genomatix Bibliosphere was used to generate a list of the 124 documented physical and functional interactions with STAT3 in diverse systems (Table S5). Of those, 26 genes showed significant regulation in the moderate and severely wasted samples (Figure 5A).

We also asked whether STAT3 target genes were also induced. Examination of the literature resulted in identification of 186 validated STAT3 target genes identified in several different species and systems (Table S6) [41], [42], [43], [44], [45], [46]. Of those, 39 were expressed in either moderate or severe cachexia or both (Figure 5B).

Among the known STAT3 targets increased in quadriceps in moderate and severe cachexia were STAT3 itself and the transcription factor CCAAT/enhancer-binding protein δ (C/EBP δ) [47]. Both transcription factors participate in induction and maintenance of acute phase response gene expression, including fibrinogen, serum amyloid A, haptoglobin, and lipid binding protein [47]–[48] [22]. C/EEBPδ has also been shown to induce expression of myostatin [49], a negative regulator of muscle mass [3], [50]. Microarray results were confirmed by qPCR and demonstrated robust induction of STAT3, C/EBPδ and acute phase response genes in moderate and severe cachexia. STAT3 also is known to induce expression of its own feedback inhibitor, Suppressor of Cytokine Signaling-3 (SOCS3). Indeed, SOCS3 was increased at the mRNA level in cachexia (Figure 5C).

**Figure 6:**
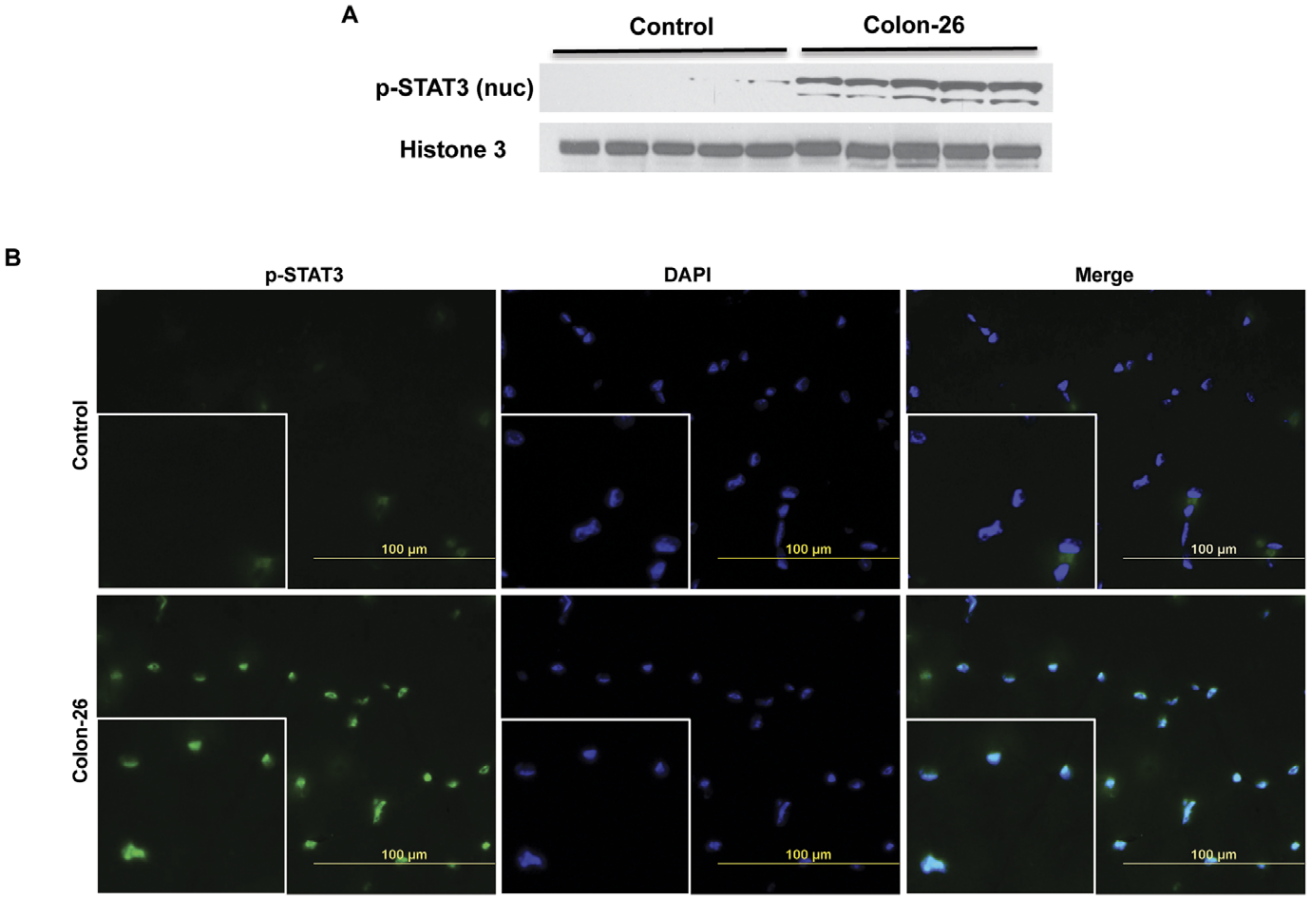
STAT3 nuclear localization is increased in the muscle of C26-bearing mice. A, p-STAT3 protein levels are increased in nuclear extracts prepared from quadriceps of severely cachectic C26 tumor-bearing mice compared to controls. n = 5 per group. B, Immunofluorescence analysis performed reveals increased pSTAT3 localization in myonuclei of gastrocnemius from severely cachectic C26 tumor-gearing mice (green). Nuclear staining is shown in blue (DAPI).

All this robust STAT3 target gene expression suggested that STAT3 activity was increased in cachexia. STAT3 is activated in part by phosphorylation at Y705, which induces dimerization, nuclear translocation and DNA binding [51], [52]. Western blotting analysis of muscle extracts showed increased pY705-STAT3 levels in both quadriceps and gastrocnemius of mice bearing the C26 tumor, in both moderate and severe cachexia (Figure 5 D). Analogously, a marked increase in pY705-STAT3 levels was observed in the liver (Figure 5 D–F). Interestingly, SOCS3 was found to be only minimally up regulated at the protein level in moderate cachexia in quadriceps and not at all in quadriceps in severe cachexia, while it was down-regulated in gastrocnemius samples in both moderate and severe cachexia (Figure 5 E–G). In contrast, SOCS3 was unchanged in liver samples from both control and cachectic mice (Figure 5 E–G). Consistent with increased muscle pY705-STAT3 levels, overall increased nuclear pSTAT3 levels and myonuclei localization were observed in muscle from mice with C26 cachexia (Figure 6A–B).

### Skeletal muscle is a major contributor for acute phase response production

**Figure 7:**
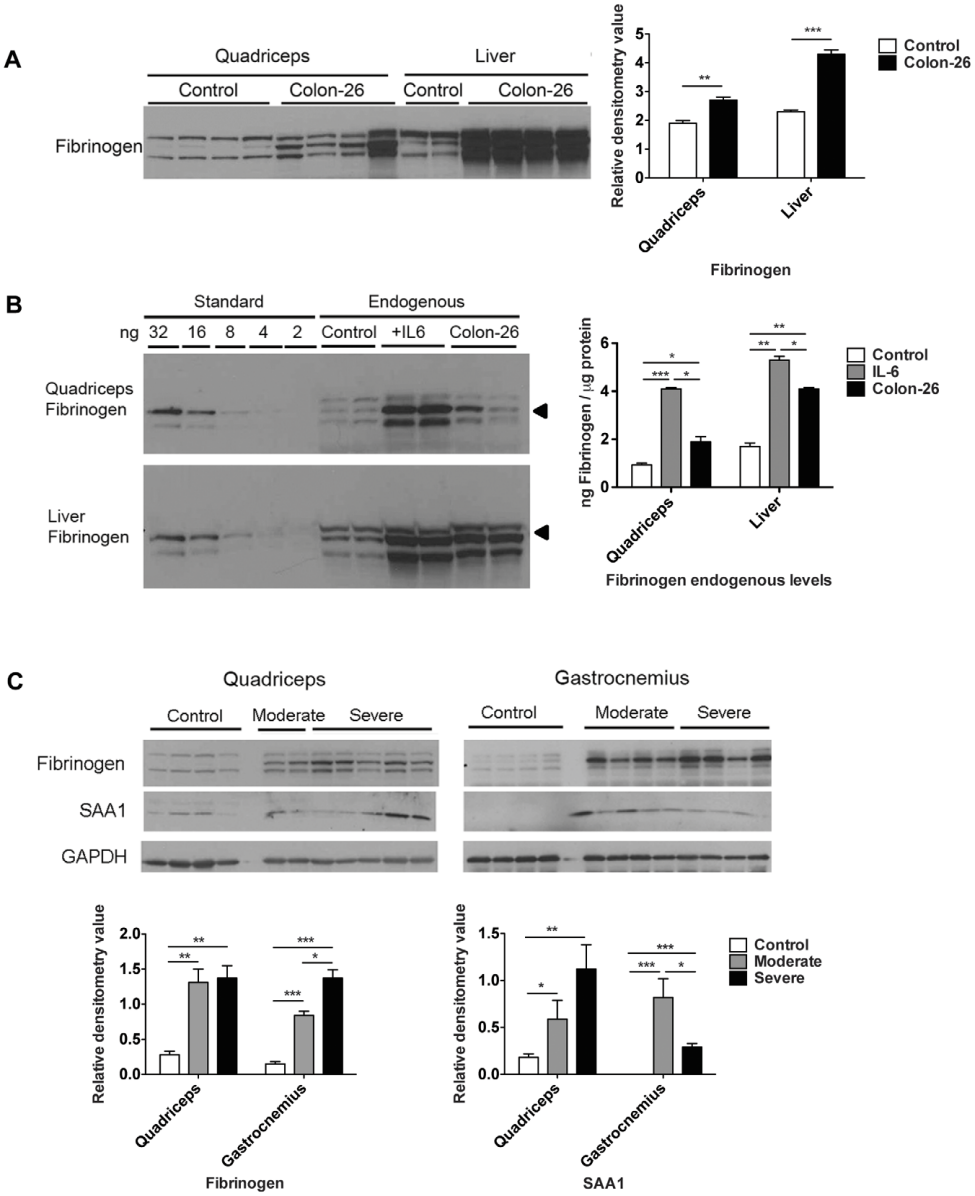
Robust expression of acute phase response proteins in skeletal muscle versus liver in C26 cachexia. A. Western blotting and quantitation of fibrinogen levels in control and C26 quadriceps and liver. Data (mean ± SEM) are expressed as relative densitometry value. **P<0.01, ***P<0.001. B, Western blotting analysis of fibrinogen standard proteins and quadriceps and liver extracts for control, CHO-IL6 injected nude mice and C26 injected CD2F1 mice. Quantitation was performed on the band indicated by the arrow. Data (means ± SEM) are expressed as ng fibrinogen / µg protein. *P<0.05, **P<0.01, ***P<0.001. C, Western blotting analysis demonstrates significantly increased fibrinogen and SAA1 protein levels in quadriceps and gastrocnemius in moderate and severe C26 cachexia. *P<0.05, **P<0.01, ***P<0.001.

Given the robust induction of acute phase gene RNA in cachexia (Figure 5), we sought to confirm expression at the protein level. By Western blotting analysis, levels of the secreted protein fibrinogen in quadriceps extracts were increased 2–12 fold in mice with C26 cachexia versus controls, depending upon which band is quantified (Figure 7A). In liver, fibrinogen protein levels were increased 2–5 fold (Figure 7A). Measuring fibrinogen signal against a standard curve of purified murine fibrinogen, normal quadriceps contained approximately 0.9 ng fibrinogen per µg of protein, while quadriceps in C26 cachexia contained 1.9 ng/µg (Figure 7B), a ∼2-fold increase. Normal liver contained 1.8 ng/µg fibrinogen, while liver in C26 cachexia contained 5.2 ng/µg fibrinogen, a ∼3-fold increase. Fibrinogen expression was also increased in quadriceps as well as liver in response to administration of IL-6 (Figure 7B), suggesting this may be a general response to conditions of high IL-6 and not limited to cancer.

These results in Figure 7A and 7B indicate that the magnitude of fibrinogen induction and synthesis in quadriceps is similar to that in the liver. Fibrinogen expression was also increased in gastrocnemius in moderate and severe cachexia (Figure 7C), indicating that fibrinogen is likely widely expressed in skeletal muscle. Protein levels of SAA1 were also increased in quadriceps and gastrocnemius by Western blotting analysis, suggesting that skeletal muscle might express proteins for most or all of the acute phase response genes induced at the mRNA level.

### Cultured skeletal muscle fibers express acute phase response proteins in response to IL-6 or STAT3 activation

**Figure 8:**
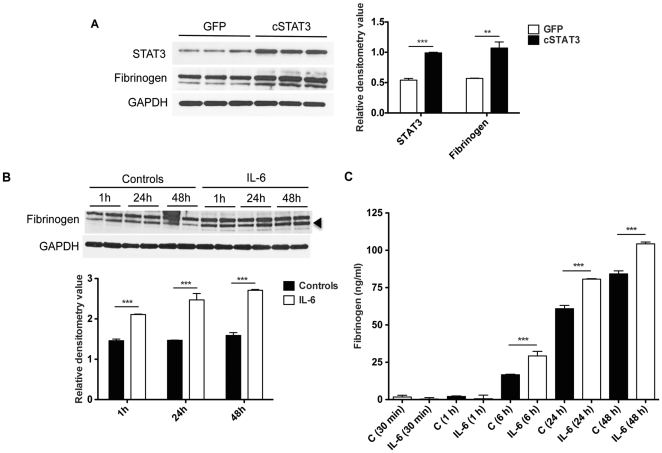
Fibrinogen is produced and released by the skeletal muscle following activation of the IL-6/STAT3 pathway. A, Western blotting analysis and quantitation of fibrinogen in C2C12 myotubes infected with Ad-cSTAT3-GFP or Ad-GFP as control. Fibrinogen expression was increased consistent with the increase in the levels of STAT3. **P<0.01, ***P<0.001 vs. GFP. B, Western blotting analysis and quantitation of fibrinogen expression in C2C12 treated with IL-6 (100 ng/ml) for 1, 24, 48 h. GAPDH was used as loading control. Increased expression of fibrinogen was observed at each time point after IL-6 treatment. Data (means ± SEM) are expressed as relative densitometry value. ***P<0.001 vs. respective controls. C, Fibrinogen levels by ELISA of the conditioned medium of C2C12 exposed to IL-6 for 30 min, 1, 6, 24, 48 h. Fibrinogen levels were significantly elevated after 6, 24 and 48 h of IL-6 treatment. Data (means ± SEM) are expressed as ng/ml. **P<0.01, ***P<0.001 vs. controls (C).

Despite the induction of fibrinogen and SAA mRNA in muscle, the protein levels of acute phase proteins in skeletal muscle extracts theoretically could be due to contaminating plasma. In order to further test our hypothesis that fibrinogen is produced directly from skeletal muscle following activation of the IL-6/STAT3 pathway, we infected C2C12 murine myotube cultures with a recombinant adenovirus expressing a constitutively activated form of STAT3, cSTAT3 [53], along with GFP as a marker. Western blotting of C2C12 extracts 48h after infection demonstrated significant elevation of fibrogen in Ad-cSTAT3-GFP cultures versus Ad-GFP cultures (+86% vs. GFP, p<0.01; Figure 8A). In order to determine whether fibrinogen was produced after IL-6 challenging, C2C12 myotubes were exposed to murine recombinant IL-6 for up to 48 h. This resulted into an overall increase in fibrinogen, both in the cellular compartment by Western blotting (Figure 8B) and in the culture medium by ELISA (Figure 8C). These experiments show that even in the absence of other cell types and tissues, skeletal muscle cells respond to IL-6 and activation of STAT3 by synthesizing acute phase protein RNAs and proteins for secretion.

## Discussion

We sought to mimic the high serum IL-6, acute phase response and muscle wasting of patients with cancer cachexia. We chose C26 adenocarcinoma, which exhibits increased circulating levels of IL-6 that coincide with muscle wasting [15]. Certain clones of the C26 that do not cause cachexia coincidently do not produce IL-6 [54], [55], [56]. Consistent with a causative role in muscle wasting in humans, circulating IL-6 has been reported to be a marker of weight loss in patients afflicted by various forms of cancer [19]. Moreover, direct administration of IL-6 to mice induces systemic muscle wasting. The consistency of such muscle wasting across various routes of IL-6 administration, including by direct injection of recombinant IL-6 [11], by transgenesis [57], by implantation of osmotic pump delivering recombinant IL-6 [13], by injection of IL-6 expressing CHO cells into athymic nude mice [12]–[13], and by transfection of plasmid DNA encoding IL-6 [14], [58], testifies to the potency by which IL-6 causes the cachectic phenotype.

Regardless, IL-6 is likely not the only cytokine mediating muscle wasting in cancer or even in the C26 model. Inhibition of IL-6 only partially rescues muscle wasting in the C26 model, causing some to conclude that IL-6 is only one of several players involved in the C26 model and cannot by itself induce the full cachectic syndrome [16]. The other cytokines we observed increased in C26 mice that might also play a role in muscle wasting include IL-6-family ligands such as LIF, as well as TNF-a, IFN-gamma, all of which can induce cachexia independently. Zhou et al. recently hypothesized IL-6 might serve only a marginal role in cachexia [59]. Consistent with prior studies [60], they reported that myostatin inhibition using a soluble receptor-fusion protein developed by Lee et al. [61] reduced muscle loss in the C26 model. Serum IL-6 levels in such mice were not different from control treated C26 mice. As well, the authors were unable to induce muscle loss in mice given recombinant IL-6 by osmotic pump. They conclude that muscle depletion in cancer cachexia might depend upon myostatin and related ligands rather than on pro-inflammatory cytokines alone. An alternative interpretation of those data, however, is that myostatin inhibition results in muscle hypertrophy, which balances IL-6 or other cytokine-induced muscle wasting. Secondly, it is also likely that the authors failed to achieve a sufficient dose of IL-6 to effect wasting. They used human IL-6, which has a 5- to 10-fold lower activity than murine IL-6 on murine cells [62], at levels 40-60-fold less than those reported to induce severe wasting [13]. (The ED_50_ for recombinant human IL-6 in the T1165.85.2.1 mouse plasmacytoma assay is 0.2–0.8 ng/ml, while it is 0.02–0.06 ng/ml for recombinant murine IL-6.) Thus the preponderance of evidence indicates that IL-6 can cause muscle wasting. Whether the effect is direct or indirect is still unclear and what mechanism leads to muscle wasting is still unknown.

Here we document that the STAT3 pathway is activated in skeletal muscle in C26-bearing mice and that expression of STAT3 target genes including the acute phase response genes are activated. Among the STAT3 target genes significantly induced was SOCS3, a classical feedback inhibitor of STAT3 activation. STAT3 induces expression of SOCS3 which binds to activated JAKs and receptors to inhibit STAT3 activation in at least three ways: by preventing binding of STAT to activated receptors, by binding and inhibiting activated JAKs and by targeting JAKs and receptors for degradation [63]. Thus high levels of SOCS3 protein should inhibit STAT3 activation. In contrast to the high SOCS3 mRNA levels in muscle, however, we observed little to no increase in SOCS3 protein either in muscle or in liver. This lack of SOCS3 protein explains in part how high pSTAT3 levels could persist regardless of high SOCS3 RNA levels and how sustained STAT3 activation might continue to drive muscle wasting while ostensibly activating its inhibitor.

Emerging data indicate that SOCS3 is regulated transcriptionally, but also post-transcriptionally and post-translationally. TNF stabilizes SOCS3 mRNA elicited by lipopolysaccharide [64], [65]. N-terminal truncated splice variants of SOCS3 generated under stress conditions show greater stability than full-length, revealing an important role for transcriptional control of SOCS3 [66]. Furthermore, Jak-mediated phosphorylation of SOCS3 at two tyrosine residues in the conserved SOCS box, Tyr204 and Tyr221, fully destabilizes SOCS3 protein and activates its proteasome-mediated degradation, while, on the contrary, a phosphorylation-deficient mutant of SOCS3, Y204F/Y221F, can remain stable in the presence of activated Jak2 and receptor tyrosine kinases [67], [68]. These results indicate that JAK/STAT activation drives not only SOCS3 mRNA expression, but also its proteolytic degradation. Recently, a reduction in SOCS3 levels has been found to be associated with, and even preceding, a decrease in MyHC in an experimental model of muscle unloading [69]. Taken together, these observations suggest that SOCS3 may not be present in sufficient quantities to inhibit STAT3 activation in cachectic muscle. Thus stabilization of SOCS3 protein might represent an intervention for muscle wasting with high IL-6.

In this experimental work we also confirm that at least two acute phase response proteins, fibrinogen and SAA1 are expressed in muscle, and that in the case of the former, at levels about half of that expressed in the liver. The significance of these results is at least three-fold. First, they establish skeletal muscle as an important source of acute phase protein synthesis. Second, they establish a molecular link between the observations of high IL-6, increased acute phase response proteins and muscle wasting in cancer. Third, they suggest a molecular mechanism through which STAT3 might causally influence muscle wasting by altering the profile of genes expressed and mRNAs translated in muscle.

Generally, the acute phase response is considered to be hepatic in origin, although several reports document expression of acute phase response genes in lung and mammary tissue [70]–[71]. Our results indicate that skeletal muscle may be a major physiological source of acute phase response proteins, both at baseline and in pathological conditions of high IL-6, including cancer. Skeletal muscle constitutes ∼40% of total body weight, while the liver is 10-fold smaller, at 4.5-5%. If the relationship of fibrinogen content we observed is representative of other acute phase response proteins, muscle might be the greater source of acute phase proteins, synthesizing about 5-times the protein produced in the liver. Thus skeletal muscle might be a key player in innate immunity.

In addition to its functional and metabolic roles, skeletal muscle is the major protein reservoir in the body. Under disease conditions, the mobilized free amino acids can also be utilized for metabolism of vital organs such as the liver, heart, brain or lung [72]. Highly plastic, skeletal muscle proteolysis has been proposed to be the main source for free amino acids for the hepatic acute phase response. Prolonged synthesis of acute phase response proteins, such as fibrinogen, is metabolically expensive and might induce nutritional deprivation of skeletal muscle [25]. Calculation by others suggest that catabolism of 2.6 grams of muscle protein is required to produce 1 gram of fibrinogen [25], [29]. As our experiments suggest, in addition to providing amino acids to the liver for production of acute phase response proteins, the skeletal muscle itself synthesizes acute phase response proteins. Indeed, we have shown the production and the release of fibrinogen from muscle *in vitro* in C2C12 myotubes following activation of the STAT3 signaling pathway. STAT3 is the main *in vivo* inducer of hepatic acute phase response expression [73], thus STAT3 is a strong candidate for mediating the muscle acute phase response.

Inflammation and correspondingly increased acute phase response protein levels are a hallmark of cancer cachexia. It has been hypothesized that hepatic synthesis of positive acute phase response proteins using amino acids liberated from skeletal muscle proteins is a major driver of skeletal muscle proteolysis, although the nature of the signal mediating both processes was not suggested [25]. Our findings show that IL-6 apparently mediates both the hepatic and skeletal muscle acute phase response through STAT3 activation in cancer, thereby positioning the IL-6/STAT3 pathway as a potentially important target to reduce skeletal muscle wasting. STAT3-mediated production of acute phase response proteins could represent a major re-prioritization of protein synthesis in skeletal muscle, away from structural proteins and towards secreted proteins. Given the enhanced proteolysis of structural proteins in cachexia, freed amino acids may be synthesized into acute phase response proteins. Ultimately this would drain protein reserves in skeletal muscle and thus represents a causal mechanism for muscle wasting in cancer.

STAT3 activation has also been observed in muscle in other experimental models of cancer cachexia with high IL-6, namely Apc^Min/+^ mice [74]. Extending these observations to other settings of muscle wasting and elevated IL-6 and related cytokines, STAT3 activation might also mediate muscle loss in obesity [75], advanced age or sarcopenia [76], inflammatory myopathies [77], burn [78], and other diseases.

## Methods

### Mice

All animal procedures were approved by the University of Miami Institutional Animal Care and Use Committee under protocols 08–174 and 10–071. CD2F1 mice were purchased from Charles River Laboratory. Colon26 cells (a gift from Dr. Donna McCarthy) were cultured in Advanced RPMI 1640 medium supplied with 10% fetal bovine serum and 1% penicillin/streptomycin and maintained in a 5% CO_2_, 37oC humidified incubator. Cells were passaged when sub-confluent, and 1×10^6^ cells per mouse were injected subcutaneously (5×10^5^ cells in each flank). Mice were weighed daily then euthanized under isoflurane anesthesia. Tissues were collected and weighed then snap frozen in liquid nitrogen. Quadriceps and liver samples in Figure 6 for IL-6 treatment were generated as in [13], [35], [36], [79], [80].

### Cell cultures

Murine C2C12 skeletal myoblasts (ATCC, Manassas, VA, USA) were grown in high glucose Dulbecco's Modified Eagle's Medium (DMEM) supplemented with 10% FBS, 100 U/ml penicillin, 100 mg/ml streptomycin, 100 mg/ml sodium pyruvate, 2 mM L-glutamine, and maintained at 37°C in a humidified atmosphere of 5% CO_2_ in air. For the experiments, cells were seeded at 35000/cm^2^ to obtain full confluence 24 h later. Differentiation to myotubes was induced by shifting confluent cultures to DMEM supplemented with 2% horse serum. The medium was changed every 2nd day, and within 5 days most of the cells were fused to form myotubes. Cells were then exposed to either Ad-CMV-GFP or Ad-cSTAT3-GFP (Vector Biolabs, Philadelphia, PA, USA) or treated with IL-6 (100ng/ml; R&D Systems, Minneapolis, MN, USA) for up to 48 h. Samples were then collected after each time point and used for further analyses.

### ELISA

Aliquots (150 µl) of culture supernatant from C2C12 myotubes exposed to IL-6 were collected at every time point. Fibrinogen levels were then assayed using the AssayMax Mouse Fibrinogen ELISA kit (AssayPro, St. Charles, MO, USA) following the instructions provided by the manufacturer.

### Serum Analyte Profiling

All samples were stored at −80°C until tested (Rules Based Medicine, Austin, TX). Platelet poor plasma samples were thawed at room temperature, vortexed, spun at 13,000 x g for 5 minutes for clarification and 150 µL was removed into a master microtiter plate. Each sample was introduced into the capture microsphere multiplexes of the RodentMAP 2.0, thoroughly mixed and incubated at room temperature for 1 hour. Multiplexed biotinylated reporter antibodies were then added, mixed and incubated for an hour at room temperature. Multiplexes were developed using an excess of streptavidin-phycoerythrin solution. Analysis was performed in a Luminex 100 instrument and the resulting data stream was interpreted using proprietary data analysis software developed at Rules-Based Medicine and licensed to Qiagen Instruments. Unknown values for each of the analytes localized in a specific multiplex were determined using 4 and 5 parameter, weighted and non-weighted curve fitting algorithms included in the data analysis package.

### RNA extraction and quantitative Real-Time PCR (qRT-PCR)

Total RNA was extracted from flash frozen quadriceps using TRIzol as previously described [81], [82]. Total RNA was quantified with a Nanodrop 8000 Spectrophotometer (Thermo Scientific, Wilmington) and its quality was assessed with a Bioanalyzer 2100 using the RNA 6000 Nano kit (Agilent, Santa Clara, CA). First-strand cDNA was synthesized from total RNA using the SuperScript First-Strand Synthesis System with SuperScript II reverse transcriptase, according to the manufacturer protocols (Invitrogen, Carlsbad, CA). The generated cDNA was used as a template in real-time PCR reactions with QuantiTect SBR-Green PCR master-mix (Bio-RAD) and were run on a Bio-Rad MyIQ machine. Quantitative Real-time PCR reactions consisted of 1x SybrGreen Supermix (Bio-Rad), 0.25 mmol/L forward and reverse primers, and 10 ng cDNA. Cycling conditions consisted of a three-step amplification and melt curve analysis using the iQ5 Real-time PCR detection System (Bio-Rad). Relative gene expression was normalized by dividing the specific expression value (starting quantity, ng) by the glyceraldehyde-3-phosphate dehydrogenase (GAPDH) expression value and calculated using the 2^-δΔCT^ method [83]. Primer sequences are provided in Table S1.

### Microarray

Biotinylated cRNA was prepared using the Illumina TotalPrep RNA Amplification Kit (Ambion, Inc., Austin, TX) according to the manufacturer's instructions, starting with 400 ng total quadriceps RNA. Successful cRNA generation was checked using the Bioanalyzer 2100. Samples were added to the BeadChip after randomization using the randomized block design to reduce batch effects. Hybridization to the MouseWG-6 v2.0 Expression BeadChips (Illumina, Inc., San Diego, CA), washing and scanning were performed according to the Illumina BeadStation 500 manual (revision C). The resulting raw microarray data were generated using Illumina BeadStudio. GeneSpring GX 7.3 was used for data normalization, statistical analysis (ANOVA, t-test) and hierarchical clustering. Only genes that were detected present (Illumina detection call p<0.01) in at least one group (control, moderate cachexia or severe cachexia) were included in the analysis. NextBio Professional and GeneGo Metacore were used for gene and pathway analysis. Genomatix Bibliosphere was used to generate a list of STAT3 associated genes.

All microarray data are MIAME compliant and have been deposited in the Gene Expression Omnibus (GEO) Database (NCBI) as Series GSE24112. Reviewers can access the data anonymously at the following link: http://www.ncbi.nlm.nih.gov/geo/query/acc.cgi?token=bpyvjkkekkwswxk&acc=GSE24112.

### Western Blotting

Total protein extract was obtained by homogenizing either skeletal muscle or C2C12 myotube samples in RIPA buffer (150 mM NaCl, 1.0% NP-40, 0.5% sodium deoxycholate, 0.1% SDS, and 50 mM Tris, pH 8.0) added with a protease inhibitor cocktail (Roche, Indianapolis, IN, USA). Nuclear extracts resulted from homogenization of muscle tissue in ice cold 10 mM HEPES, pH 7.5, containing 10 mM MgCl_2_, 5mM KCl, 0.1 mM EDTA pH 8.0, 0.1% Triton X-100, 0.1 mM phenylmethanesulfonyl fluoride [PMSF], 1 mM DTT, 2 µg/ml aprotinin, 2 µg/ml leupeptin. Samples were then centrifuged (5 min, 3000 g), pellets resuspended in ice cold 20 mM HEPES, pH 7.9, containing 25% glycerol, 500 mM NaCl, 1.5 mM MgCl_2_, 0.2 mM EDTA, pH 8.0, 0.2 mM PMSF, 0.5 mM DTT, 2 µg/ml aprotinin, 2 µg/ml leupeptin, and incubated on ice for 30 min. Cell debris were removed by centrifugation (5 min, 3000 g) and the supernatant collected and stored at –80°C. Protein concentration (for both total and nuclear extracts) was determined using the Bradford protein assay method (Thermo Fisher Scientific, Suwanee, GA, USA).

Either total or nuclear protein extracts (30 µg) were then electrophoresed in gradient SDS gels. Gels were transferred to nitrocellulose membranes. Membranes were blocked with 1X TBS, 0.1% Tween-20 (TBST) with 5% w/v Bovine Serum Albumin (BSA) at room temperature for 1 hour, followed by an overnight incubation with diluted antibody in blocking buffer at 4°C with gentle shaking. After washing with TBST, the membrane was incubated at room temperature for 1 hour with a goat polyclonal anti-rabbit IgG secondary antibody conjugated to horseradish peroxidase (HRP) (sc-2313, Santa Cruz Biotechnology Inc., Santa Cruz, CA). Membranes were visualized with enhanced chemiluminescence (Pierce SuperSignal Pico or Femto) followed by exposure to film. Antibodies were pSTAT3 (9145), STAT3 (9132), GAPDH (2118), Histone H3 (4499) from Cell Signaling (Beverly, MA), and SOCS3 (Abcam 3693), Fibrinogen (Dako A0080), and SAA1 (R & D Systems AF2948). Mouse fibrinogen for quantitation was from Oxford Biomedical Research.

### Immunofluorescence

Cryosections (8 µm) from gastrocnemius muscles of both controls and C26-bearing mice were fixed in 3% formaldehyde, permeabilized in PBS-Triton 0.1% and incubated with pSTAT3 primary antibody (9145, Cell Signaling) overnight at 4°C. After three washes in PBS (5 min each), sections were incubated with the fluorescent secondary antibody (Alexa Fluor 488, Invitrogen, Carlsbad, CA, USA) in common antibody diluent (BioGenex, San Ramon, CA, USA) for 1 h at room temperature (RT) in the dark. After being washed three times with PBS (5 min each), sections were counterstained with DAPI and mounted with ProLong Gold Antifade mounting medium (Invitrogen, Carlsbad, CA, USA). Images were collected with constant exposure time across samples.

### Statistical analysis

All results were expressed as means ± SEM. Representative Western blots show independent samples. Quantitation of the band intensities was performed using the ImageJ software (US National Institutes of Health, Bethesda, MD, USA). Significance of the differences was evaluated by analysis of variance (ANOVA) followed by Tukey's test for experiments with more than 2 groups or by Student t-test between 2 groups.

## Supporting Information

Table S1 **Primer sequences for real-time quantitative RT-PCR.**
(DOC)

Table S2 **Genes regulated in both moderate and severe cachexia.**
(XLS)

Table S3 **Genes regulated only in moderate C26 cachexia.**
(XLS)

Table S4 **Genes regulated only in severe C26 cachexia.**
(XLS)

Table S5 **Documented physical and functional interactions between STAT3 and other genes, by Genomatix.**
(XLS)

Table S6 **Validated STAT3 target genes, based upon the literature.**
(XLS)
