# Supplementary material for: STAT3 Activation in Skeletal Muscle Links Muscle Wasting and the Acute Phase Response in Cancer Cachexia
Source: PLoS One. 2011 Jul 20;6(7):e22538. doi: 10.1371/journal.pone.0022538 (PMC3140523; doi:10.1371/journal.pone.0022538)
Supplement: Table S1 — Primer sequences for real-time quantitative RT-PCR. (DOC) [file pone.0022538.s001.doc]

| | **Table S1: Primer sequences for real-time quantitative RT-PCR .** | | | | | --- | --- | --- | --- | | **Gene Symbol** | **Tm** | **Primers** | **Amplicon (bp)** | | FBXO32 | 63°C | S: 5’-CCATCAGGAGAAGTGGATCTATGTT-3’ | 75 | |  |  | AS: 5’-GCTTCCCCCAAAGTACAGTA-3’ |  | | SOCS3 | 62°C | S: 5’-TGCAGGAGAGCGGATTCTAC-3’ | 132 | |  |  | AS: 5’-TGACGCTCAACGTGAAGAAG-3’ |  | | PTPN2 | 65°C | S: 5’-CATTTCTGGCTCATGGTGTG-3’ | 141 | |  |  | AS: 5’-CACACTGAATCCCGTTTCCT-3’ |  | | GAPDH | 65 °C | S: 5’-TGCACCACCAACTGCTTAG-3’ | 147 | |  |  | AS: 5’-GGATGCAGGGATGATGTTC-3’ |  | | CEBPD | 65°C | S: 5’-ATCGACTTCAGCGCCTACAT-3’ | 101 | |  |  | AS: 5’-GCTTTGTGGTTGCTGTTGAA-3’ |  | | STAT3 | 65°C | S: 5’-TGAAGGTGGTGGAGAACCTC-3’ | 123 | |  |  | AS: 5’-GCTGCTGCATCTTCTGTCTG-3’ |  | | SAA1 | 62°C | S: 5’-GCGAGCCTACACTGACATGA-3’ | 119 | |  |  | AS: 5’-TTTTCTCAGCAGCCCAGACT-3’ |  | | LBP1 | 65°C | S: 5’-GGCTGCTGAATCTCTTCCAC-3’ | 109 | |  |  | AS: 5’-TAAGGCTGCAGGTCAGAGGT-3’ |  | | FGG | 65°C | S: 5’-GTACGTGGCCCAAGAGGTTGT-3’ | 145 | |  |  | AS: 5’-TAAAATCCTGGTTGGCTTCG-3’ |  | | HP | 65°C | S: 5’-GTATGTCATGCTGCCTGTGG-3’ | 129 | |  |  | AS: 5’-CAGAAGGTGTGCTCGTTCAA-3’ |  | | ACTC1 | 58 °C | S: 5`-CCAGCCCAGCTGAATCCA-3’ | 156 | |  |  | AS: 5`-ACACATCCTGGCACAGCTTTG-3’ |  | | TRIM63 | 65°C | S: 5’-ACCTGCTGGTGGAAAACATC-3’ | 147 | |  |  | AS: 5’-AGGAGCAAGTAGGCACCTCA-3’ |  | | *Tm, melting temperature.* | |  |  | |
| --- | --- | --- | --- | --- | --- | --- | --- | --- | --- | --- | --- | --- | --- | --- | --- | --- | --- | --- | --- | --- | --- | --- | --- | --- | --- | --- | --- | --- | --- | --- | --- | --- | --- | --- | --- | --- | --- | --- | --- | --- | --- | --- | --- | --- | --- | --- | --- | --- | --- | --- | --- | --- | --- | --- | --- | --- | --- | --- | --- | --- | --- | --- | --- | --- | --- | --- | --- | --- | --- | --- | --- | --- | --- | --- | --- | --- | --- | --- | --- | --- | --- | --- | --- | --- | --- | --- | --- | --- | --- | --- | --- | --- | --- | --- | --- | --- | --- | --- | --- | --- | --- | --- | --- | --- | --- | --- | --- | --- |
